# Non-Contact Measurement of the Surface Displacement of a Slope Based on a Smart Binocular Vision System

**DOI:** 10.3390/s18092890

**Published:** 2018-08-31

**Authors:** Leping He, Jie Tan, Qijun Hu, Songsheng He, Qijie Cai, Yutong Fu, Shuang Tang

**Affiliations:** 1 School of Civil Engineering and Architecture, Southwest Petroleum University, Chengdu 610500, China; 201231010028@swpu.edu.cn (L.H.); 201722000532@stu.swpu.edu.cn (J.T.); sheng583786602@163.com (S.H.); 201622000204@stu.swpu.edu.cn (Y.F.); 201722000537@stu.swpu.edu.cn (S.T.); 2 School of Transportation and Logistics, Southwest Jiaotong University, Chengdu 610031, China; caiqijieswjt@my.swjtu.edu.cn

**Keywords:** slope monitoring, surface deformation, binocular vision, subpixel resolution, user defined target

## Abstract

The paper presents an intelligent real-time slope surface deformation monitoring system based on binocular stereo-vision. To adapt the system to field slope monitoring, a design scheme of concentric marking point is proposed. Techniques including Zernike moment edge extraction, the least squares method, and k-means clustering are used to design a sub-pixel precision localization method for marker images. This study is mostly focused on the tracking accuracy of objects in multi-frame images obtained from a binocular camera. For this purpose, the Upsampled Cross Correlation (UCC) sub-pixel template matching technique is employed to improve the spatial-temporal contextual (STC) target-tracking algorithm. As a result, the tracking accuracy is improved to the sub-pixel level while keeping the STC tracking algorithm at high speed. The performance of the proposed vision monitoring system has been well verified through laboratory tests.

## 1. Introduction

Landslide disasters cause serious damage to human life and the economy. Surface deformation is an important basis for assessing the safety status of a slope. At present, slope surface deformation monitoring methods are of five main classes: geodetic methods, global positioning system (GPS) technology, three-dimensional (3D) laser scanning, interferometric synthetic-aperture radar (INSAR) technology, and digital photogrammetry. Geodetic measurement [1] is a traditional monitoring method; however, owing to a low observation frequency and low intelligence, it is difficult to obtain monitoring data that has spatial-temporal continuity. GPS [2] has a high degree of intelligence and can achieve full-time monitoring; however, its target setting is limited, that is, no obstacles are allowed within a range of 15° around the elevation angle of the station in most cases [3]. Both 3D laser scanning [4] and INSAR technology [5] are free at the selection of the marker points, but are costly and difficult to apply to slopes covered with vegetation.

In view of the above problems, owing to their non-contact and cost-effective features, vision-based digital photogrammetry systems have been studied extensively in recent years. The method converts the image coordinates into spatial coordinates by tracking the target image, and obtains the structural deformation information [6]. In practice, the most prominent limitation of visual sensor systems is the measurement accuracy. The main factors affecting this accuracy are (1) marker points and (2) target tracking and positioning. On the one hand, some scholars have used natural marking points when applying machine vision technology to structural deformation monitoring. For instance, Yoon et al. [7] use the Harris corner detection algorithm to extract the feature points of the specified area of a structure. Khuc et al. [8] use a Hessian matrix [9,10] to extract the key points on a steel beam. At the same time, others directly use obvious features such as a light-emitting diode lamps [11] and structural bumps [12,13,14] as the monitoring markers. On the other hand, target markers with specially designed features, such as a circle [15,16,17], a checkerboard [18,19,20], or a random pattern [21], have also been widely used. The position of a feature can be detected and then transformed into the coordinate information. Considering the insufficient feature points of a large slope, it is necessary to artificially set the landmarks to achieve a measurement. The positioning accuracy of the landmarks greatly determines the accuracy of the monitoring results. Common image positioning methods include the least squares fitting method [22], grey weighted centroid method [23], SUSAN algorithm [24], and Hough transform method [25]. Therefore, the authors propose a concentric marker and positioning method that adapts to the visual monitoring system applied. Even if the slope is covered by vegetation, high-precision positioning of the measuring point can be achieved.

For continuous intelligent monitoring, consumer-level cameras can be used to track and locate the landmarks in each frame. The current tracking algorithms mainly include KLT [7,26], CN [27], KCF [28], ODFS [29], and spatial-temporal contextual (STC) [30,31]. However, existing target-tracking algorithms have mostly been studied with regard to their intelligent stability, whereas a few have been studied for their positioning accuracy. To meet the accuracy requirements of deformation monitoring, scholars usually use template matching technology to obtain high-precision monitoring results. A variety of methods are applied to template matching for vision sensors including digital image correlation, pattern matching, optical flow, sub-pixel Hough transforms, random sample consensus, edge detection, sum of squared differences, scale-invariant feature transform, and the orientation code matching(OCM) [14,16,20,32,33,34,35]. Based on the OCM template matching algorithm, Feng et al. [14] demonstrated the high accuracy of the vision sensor for dense full-field displacement measurements through experimental results. Javh et al. [34] showed a sub-pixel displacement resolution of less than thousandths of a pixel by a simplified gradient-based optical flow method under laboratory conditions. However, these methods are limited in obtaining the three-dimensional deformation of a structure. It is generally known that slope surface monitoring requires three-dimensional information. Based on binocular stereoscopic vision measurement technology, to overcome the original frame-by-frame selection method for targets, we combine the temporal-spatial contextual visual tracking algorithm (STC) [31] with sub-pixel image registration technology [36], and improve the tracking accuracy to the sub-pixel level while maintaining the high speed STC algorithm to achieve real-time monitoring.

In this paper, to realise the intelligent real-time monitoring of a slope surface deformation, binocular stereo-vision measurement technology is introduced into the monitoring of the slope surface deformation, and the designs of concentric landmark points and high-precision image positioning methods are described. At the same time, the existing tracking technology is improved to achieve high-precision target tracking and spatial positioning. Finally, laboratory tests conducted to verify the validity and accuracy of the proposed method are detailed.

## 2. Proposed Smart Binocular Vision System

### 2.1. Overview

**Figure 1:**
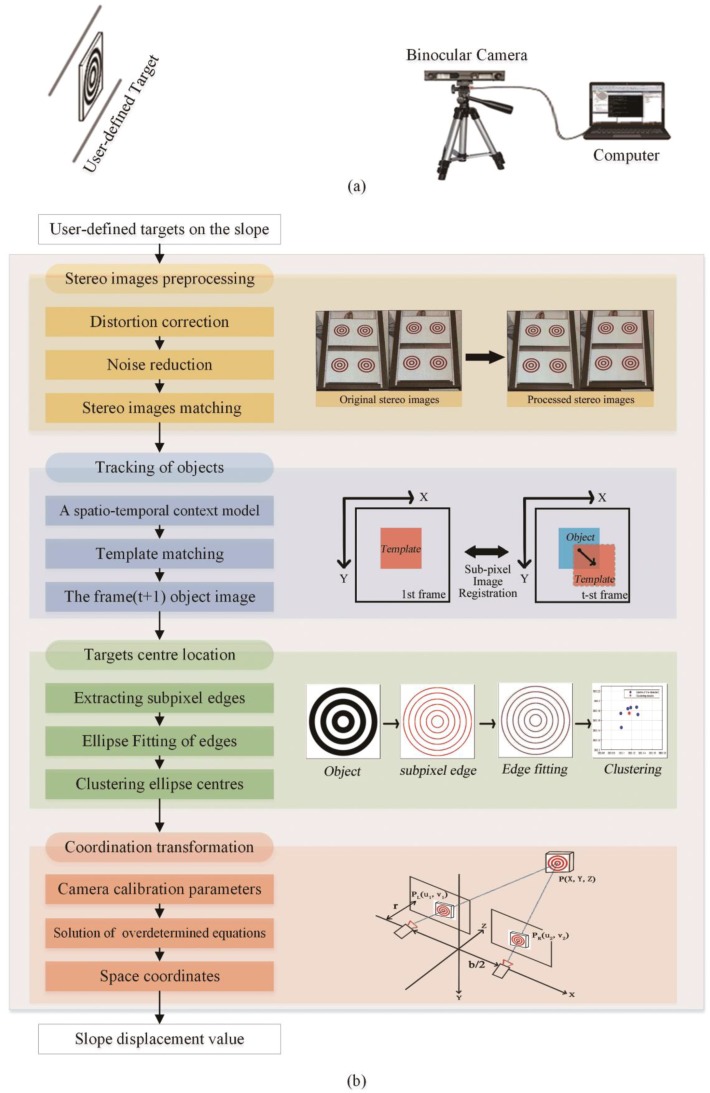
Displacement measurement system based on binocular vision technology: (**a**) hardware and (**b**) software.

A binocular vision based displacement measurement system is typically composed of hardware and software (see Figure 1). The hardware components consist of a commercial binocular camera, a computer for storing and processing data, and a custom target. The binocular camera has a zoom lens from 4 mm to 12 mm, with a maximum resolution of 2560 × 960 pixels, and an adjustable acquisition frame rate up to 60 fps. The stereo baseline can be adjusted from 4.5 cm to 18 cm, and the field of view of the camera is from 29° to 78°. The vision system was worked on a laptop (Lenovo Xiaoxinchao500, Beijing, China) with an Intel i7-7500U processor with 4 GB of RAM and a mechanical hard disk drive. The movements of a target can be recorded and tracked by the camera and synchronously transferred to the computer, where the displacement is calculated using object centre location algorithms and coordinate transformations.

### 2.2. Target Design

Visual measurement technology is based on marker imaging. It must be clear that the precise positioning of an object requires searching for obvious feature points. Natural targets are often used in low-precision or near-distance measurements, whereas artificial targets are often used in high-precision or long-distance measurements, particularly for large-scale outdoor engineering structures such as slopes. In this chapter, the design and positioning methods of existing mark points are proposed to achieve the high-precision positioning of the measuring points.

#### 2.2.1. Design Scheme

A round mark point is one of the most common forms of feature points in monitoring. However, a circle shows an elliptical shape after a perspective projection in computer vision imaging. In general, region- and edge-based technologies are used in elliptical centre positioning. The former is inefficient in terms of its operation, and has difficulty ensuring the noise removal effect, and thus it cannot adapt to the complex environment of a slope-engineering site. Instead, the latter can effectively avoid these problems [37]. Therefore, this study uses edge-based elliptical centre positioning technology.

Ellipse fitting technology has been widely used owing to its good fault tolerance, adaptive noise environment, and high efficiency in achieving centre positioning. The basis of the ellipse fitting technique is to obtain the edge information of an image. This study uses sub-pixel edge detection technology to achieve high-precision edge extraction, providing the best edge information for the centre positioning.

After obtaining the centre coordination of each circular mark, the clustering algorithm is used to gain the representative value of the centre of the circle, avoiding the influence of singular values and random errors on the centre orientation.

#### 2.2.2. Object Positioning Method

Remote monitoring requires higher accuracy. This study uses the modified template of Gao et al. [38] to extract concentric sub-pixel edges, the basic principle of which is as follows: calculate the edge parameters according to the rotational invariance of the Zernike moment, and use the edge parameters to determine whether it is an edge to accurately extract the edge position. An ellipse is then fitted using the least squares method [39] to locate the centre coordinates of each concentric circle:

(1) $$x_{c}=\frac{be-2cd}{4ac-b^{2}}\;y_{c}=\frac{bd-2ae}{4ac-b^{2}}$$

*a*, *b*, *c*, *d*, *e* are the coefficients of the general equation of the ellipse $ax^{2}+bxy+cy^{2}+dx+ey+f=0$, and *f* is a constant. Finally, the value of the centre is extracted based on the k-means clustering algorithm [40]. The basic principle lies in the optimisation of the following formula:

(2) $$J=\overset{N}{\underset{n=1}{\sum }}\overset{K}{\underset{k=1}{\sum }}r_{nk}{\left\|x_{n}-\mu_{k}\right\|}^{2}$$

In this equation, N represents the number of data samples; K is the number of clusters; $r_{nk}$ represents 1 when data point n is assigned to class k, and is 0 otherwise; $x_{n}$ indicates the sample data object; and $\mu_{k}$ is the cluster centre.

**Figure 2:**
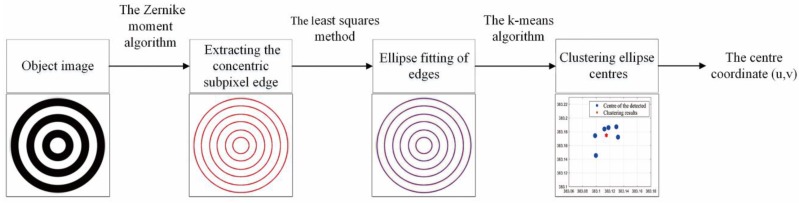
Target location process.

The method in this study achieves the function of target centre location through programming (see Figure 2).

#### 2.2.3. Target Parameter Design

(1) Number of Circles

The optimal number of concentric circles is determined based on the central location technology of the marked points described in the previous section. The test image is an idealised concentric circle of different layers with a size of 1712 × 1712 pixels, and the 2-layer concentric circles are minimal, with diameters of 10 mm and 20 mm, respectively. Then, we add circles outside the previous 2-layer concentric circles with a bigger 5 mm radius in other six patterns of concentric circles.

**Figure 3:**
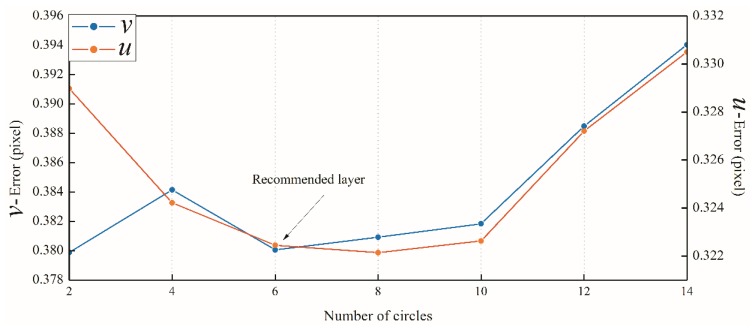
Analysis of test results of concentric circles.

As shown in Table 1 and Figure 3, errors of the target coordinates generally decrease with the increase in concentric circles and then experience a relatively stable stage at layer 6 to 10. With the continuous increase in concentric circles, errors also rise almost linearly. Considering that in field measurement, when the number of concentric circles increases, the size of the targets increases accordingly, and the image noise caused by environmental factors such as air flow will also increase. To avoid this problem and save the early time cost, this study suggests setting the number of concentric layers to six (see Figure 3). At the same time, we can see that the algorithm has a deviation of 0.4 pixels. Since this deviation is stable, the center of each positioning is almost constant, so the accuracy requirements of the measurement system are met.

(2) Minimum positioning size

At different measurement distances, the pixel sizes of the targets in the image plane of the camera are inconsistent. This study will use six concentric (ellipse) circles with different pixel sizes to obtain the minimum detectable pixel size through the above-mentioned centring location technique, and thus provide guidance for a slope monitoring landmark design.

From the positioning error analysis results (see Table 2), the positioning point is located on the upper-left side of the theoretical point when the pixel resolution is above 28 × 28 pixels. The positioning error u is between 0.34 and 0.42, and the floating range is 0.07 pixels. Meanwhile, the positioning error ν is between 0.25 and 0.40, and the floating range is 0.25 pixels. It can be seen that the center of its positioning is relatively stable. In summary, this study suggests that the concentric pixel resolution should be greater than 28 × 28 pixels to ensure its effective positioning.

#### 2.2.4. Noise Robustness

In image processing, noise is a ubiquitous phenomenon with great interference. In engineering applications, the obtained image is different from the “real” image due to the factors such as image acquisition equipment and natural environment. This part of difference is noise. In this section, the simulation noise image is used to verify the stability of the algorithm. At present, the image noise is mainly gaussian noise and salt noise.

In this study, the 6-layer concentric circle images, a size of 767 × 767 pixels, with different variance Gaussian noise and different density impulse noise were obtained by means of Matlab simulation, and then the positioning experiment was carried out. Compared with the measured values in the non-noise case, the error of the proposed algorithm under the influence of noise is calculated. Finally, the stability of the proposed algorithm in dealing with noise is verified by comparison with the gravity method [23] based on regional positioning.

**Figure 4:**
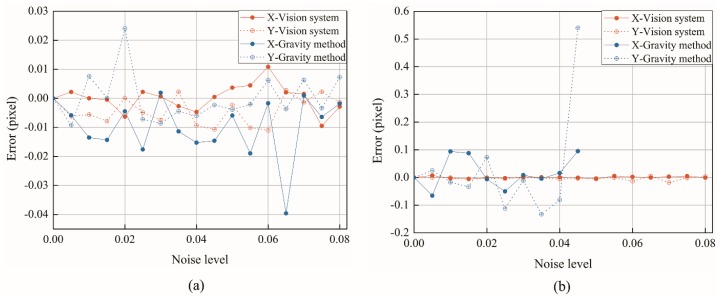
Positioning error of marker points under the influence of noise; (**a**) Considering Gaussian noise; (**b**) Considering impulse noise.

As shown in Figure 4, when we increase the two noise levels to 0.08 respectively, we can see from the error analysis results that the fluctuation of the gravity method represented by the blue curve is significantly higher than that of the red curve. Locally, the centering technique based on the gravity method has a mis-positioning point when the impulse noise density reaches 0.05, and the center positioning cannot be achieved. The algorithm proposed in this study can still achieve accurate positioning when the impulse noise density reaches 0.08, and the maximum error is only 0.0183 (see Table 3). It is proved that the concentric center positioning method proposed in this study shows better accuracy and stability when dealing with noise.

### 2.3. Target Tracking

#### 2.3.1. Theory

**Figure 5:**
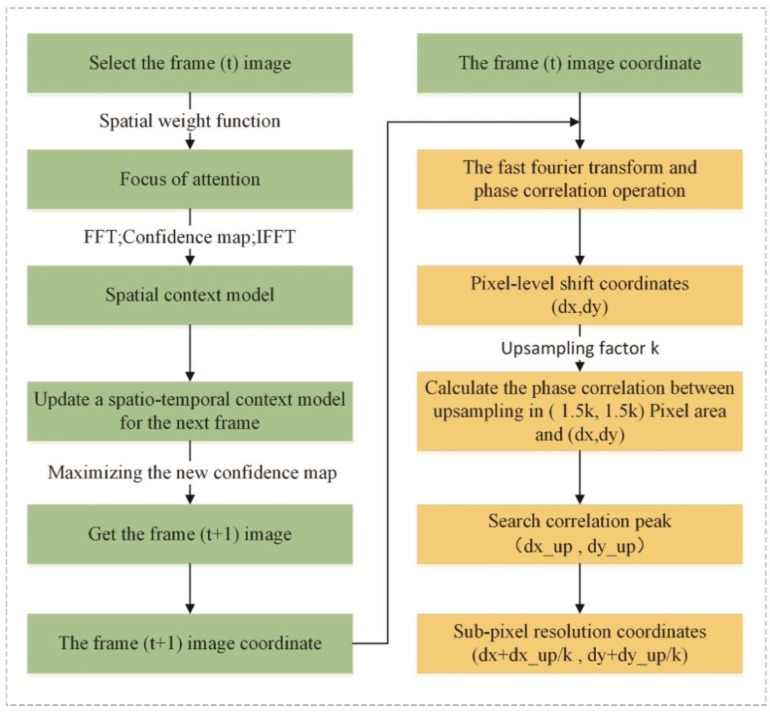
Flowchart of object tracking based on spatial-temporal contextual (STC) and Unsampled Cross Correlation (UCC).

The STC tracking algorithm and sub-pixel image registration technology are employed to improve the target tracking accuracy. Theoretically, the accuracy of this method can probably increase to the sub-pixel level while maintaining the high speed of the STC algorithm. The basic flow is shown in Figure 5.

Step 1: Target pixel-level positioning based on a confidence map. In the first frame, we suppose that the target location has been manually initialised. At the *t*-th frame, we learn the spatial context model $h^{sc}\left(x\right)$ for (3) updating the spatio-temporal context model $H_{t+1}^{stc}$ (4) and apply it to detect the object location in the (*t* + 1)-th frame. The object location $x_{t+1}^{*}$ (5) in the (*t* + 1)-th frame is determined by maximising the new confidence map.

(3) $$h^{sc}\left(x\right)=F^{-1}\left(\frac{F\left(be^{-{\left|\frac{x-x^{*}}{\alpha }\right|}^{\beta }}\right)}{F\left(I\left(x\right)\omega_{\sigma }\left(x-x^{*}\right)\right)}\right)$$

(4) $$H_{t+1}^{stc}=\left(1-\rho \right)H_{t}^{stc}+\rho h_{t}^{sc}$$

(5) $$x_{t+1}^{*}=\underset{x\in \Omega_{c}\left(x_{t}^{*}\right)}{\mathrm{argmax}}c_{t+1}\left(x\right)$$

where $c_{t+1}\left(x\right)$ is represented as

(6) $$c_{t+1}\left(x\right)=F^{-1}\left(F\left(H_{t+1}^{stc}\left(x\right)\right)⨂F\left(I_{t+1}\left(x\right)\omega_{\sigma t}\left(x-x_{t}^{*}\right)\right)\right)$$

In this function, F denotes the fast Fourier transform function, $F^{-1}$ is the inverse of F, b is a normalisation constant, α is a scale parameter, β is a shape parameter, I() is the image intensity that represents the appearance of the context, and $\omega_{\sigma }()$ is the weighted function defined by

(7) $$\omega_{\sigma }\left(z\right)=ae^{-\frac{{\left|z\right|}^{2}}{\sigma^{2}}}$$

where a is a normalisation constant, and σ is a scale parameter.

Step 2: Target sub-pixel-level location based on image registration. UCC template matching technology is used to conduct template matching between the target and template images. The cross-correlation in the neighbourhood of 1.5 × 1.5 pixels with respect to the initial estimate is calculated using the up-sampling factor k, which can achieve a 1/k registration accuracy of the pixel, eliminate the tracking drift, and allow the tracking process to reach the sub-pixel accuracy. The specific process is as follows:

We assume that the t-frame target tracking image is f(x,y), template image is g(x,y), and the amount of drift between the two images is (dx,dy).

(8) g(x,y)=f(x−dx,y−dy)

Convert the image into frequency domain using Fourier transforms:

(9) $$G\left(u,v\right)=F\left(u,v\right)*e^{-i*2\pi *\left(udx+vdy\right)}$$

Divide the above equation to obtain the cross power spectrum:

(10) $$H\left(u,v\right)=\frac{G\left(u,v\right)F^{*}\left(u,v\right)}{\left|G\left(u,v\right)\right|*\left|F^{*}\left(u,v\right)\right|}=e^{-i*2\pi *\left(udx+vdy\right)}$$

In this function, $F^{*}$ represents the complex conjugate of F. For the mutual power spectrum, the Dirac function can be obtained by inverse Fourier transform. The pixel-level registration is finally achieved by locating the peak coordinates of the Dirac function.

After achieving pixel-level registration, the pixel-level drift value of the image can be obtained, and then the sub-pixel drift coordinate extraction is implemented by using the upsampling algorithm within one pixel drift. The upsampling multiple k = 100, therefore, the registration accuracy can reach 0.01. After the image is amplified by upsampling, the image phase correlation algorithm is used to obtain the drift value of the image. Since the image drift value at this time is the result after the upsampling, it is necessary to perform the reduction in combination with the upsampling multiple, that is, multiply by 0.01 to obtain the sub-pixel drift coordinates. After obtaining the pixel-level and sub-pixel translation coordinates respectively, the final result of sub-pixel image registration can be obtained by combining the two.

In this study, the above-mentioned upsampling and image phase correlation algorithm is used to correct the drift phenomenon of the target tracking process, and then the tracking coordinates can be combined to achieve accurate target positioning. Eventually the target tracking accuracy is raised to the sub-pixel level.

#### 2.3.2. Performance Evaluation

**Figure 6:**
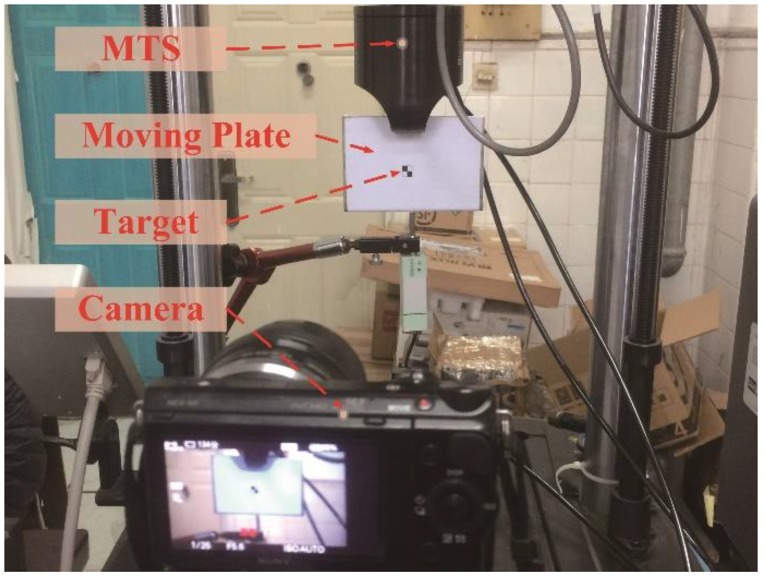
Setup for moving platform tests.

The moving platform test experiments were used to evaluate the performance of the improved STC algorithm. In this study, the MTS test machine was used to clamp the moving plate to reciprocate up and down, and it was continuously monitored by the camera. In order to better demonstrate the advantages of the improved STC algorithm, two different loading methods were set up in this study, namely linear loading and sinusoidal loading. The frequency of the MTS tester was set to 0.1 Hz and the amplitude was set to 9 mm. The moving platform test setup is shown in Figure 6.

**Figure 7:**
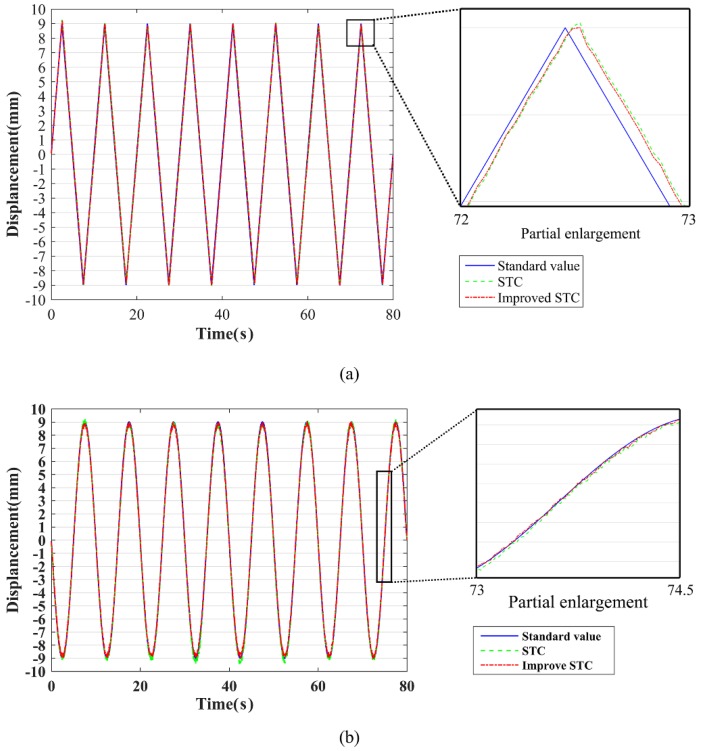
The moving platform tests results: (**a**) linear loading; (**b**) sinusoidal loading.

After obtaining the moving plate test sequence image, the target object is tracked and detected by using the STC algorithm and the improved STC algorithm. The pixel coordinate transformation is converted into physical coordinate transformation by the scale factor calculation method [41], and the result is shown in Figure 7.

As shown in Figure 7, both the STC algorithm and the improved STC algorithm can achieve target tracking measurements. From the partial enlargement, the measured value of the improved STC algorithm is less fluctuating. According to the measurement error analysis, the normalized root mean squared error (NRMSE) of the STC algorithm is 0.0127 for linear loading, and the improved STC algorithm is 0.0106. When sinusoidal loading, NRMSE of the STC algorithm measurement is 0.0122, and the improved STC algorithm is 0.0098. It can be seen that the improved STC algorithm can effectively reduce the measurement error, improve the measurement accuracy, and make the measurement result more stable and reliable.

### 2.4. Coordinate Transformation

**Figure 8:**
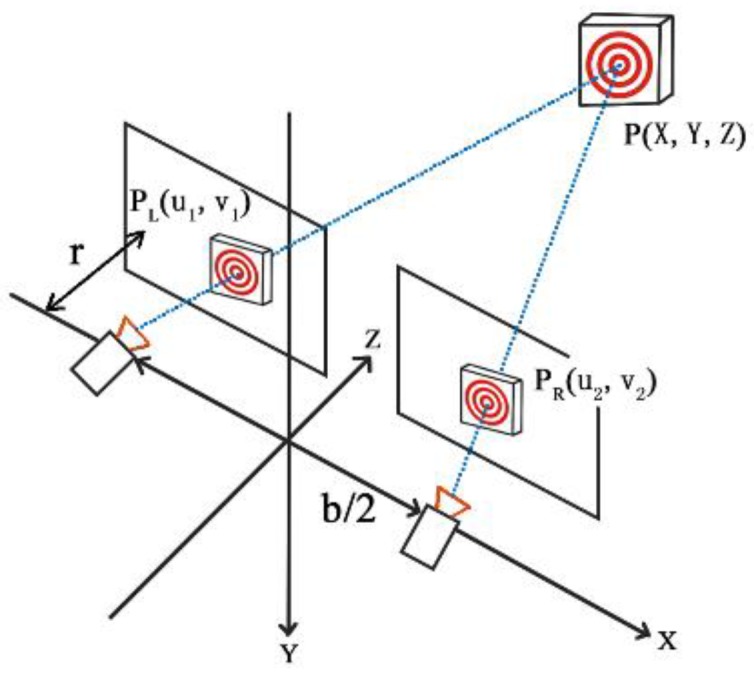
Schematic of binocular vision measurement.

According to Zhang’s calibration method [42], two coefficient matrices can be constructed by calibrating a binocular camera. The left- and right-image pixel coordinates are then combined using a coefficient matrix to solve the over-determined equations and obtain the spatial coordinates. After obtaining the spatial coordinates of the target in each frame of the image, the displacement value of the measurement point can be quantified to obtain the surface deformation of the slope. The calculation principle is shown in Figure 8.

(11) $$\left[\begin{array}{c} u_{1}\\ v_{1}\\ 1 \end{array}\right]=\left[\begin{array}{cccc} p_{00}^{1} & p_{01}^{1} & p_{02}^{1} & p_{03}^{1}\\ p_{10}^{1} & p_{11}^{1} & p_{12}^{1} & p_{13}^{1}\\ p_{20}^{1} & p_{21}^{1} & p_{22}^{1} & p_{23}^{1} \end{array}\right]\left[\begin{array}{c} X\\ Y\\ Z\\ 1 \end{array}\right]\;\left[\begin{array}{c} u_{2}\\ v_{2}\\ 1 \end{array}\right]=\left[\begin{array}{cccc} p_{00}^{2} & p_{01}^{2} & p_{02}^{2} & p_{03}^{2}\\ p_{10}^{2} & p_{11}^{2} & p_{12}^{2} & p_{13}^{2}\\ p_{20}^{2} & p_{21}^{2} & p_{22}^{2} & p_{23}^{2} \end{array}\right]\left[\begin{array}{c} X\\ Y\\ Z\\ 1 \end{array}\right]$$

## 3. In-Laboratory Validation Test

This chapter describes tests to verify the method proposed in this study.

### 3.1. Static Distance Measurement Test

**Figure 9:**
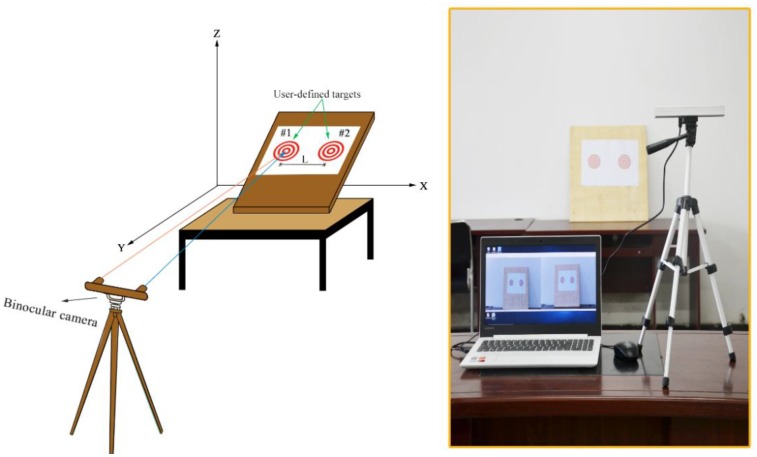
Setup for static distance measurement test, **left:** image of 3D simulation test, **right:** laboratory test scene.

To quantify the effect of the mark point size and centre distance on the measurement accuracy, two sets of tests are described in this section. The major instrumentation includes the targets, binocular cameras, and computer (see Figure 9). The stereo baseline is set to 12 cm, and the distance from the camera to the measuring point is 4 m. Then, we get the best image by manually adjusting the focus and keep it constant.

Test 1: The sizes of the marked points are different, and the centres of the circles are the same. The marker points were designed using eight different sizes according to the relevant parameters in Chapter 3. It is assumed that the minimum concentric diameter is D, the remaining diameters are $D_{n}=D\times n$, n is an integer from 1 to 6, and the distance between two centres is 150 mm accordingly, which is measured in vector drawing tool Coreldraw.

Test 2: The marked points have the same size, but the centres of the two circles are different. We chose a minimum diameter of the marker point of 15 mm, and a distance to the circle centre of 100 to 300 mm.

The results obtained after calculating the spatial coordinates using the proposed method in Chapter 2 are shown in Table 4 and Table 5.

As can be seen from the above table, during the testing of different target size measurements, the distance between the two markers was measured using a stereo-vision system. The mean value of the measurement error is 0.1923 mm, and the maximum error is 0.2367 mm. During the testing of the circle centre distance for different sign points, the average value of the measurement error is 0.2153 mm, and the maximum error is 0.2898 mm. This shows that the system can achieve millimetre level accuracy in monitoring, and ensure the accuracy of the spatial coordinate measurements. Furthermore, the development of its error has no obvious relationship with the marked point size and the distance from the centre of the circle, and thus can reach the millimetre level in any sized measurement of the mark. 

### 3.2. Moving Platform Experiment

**Figure 10:**
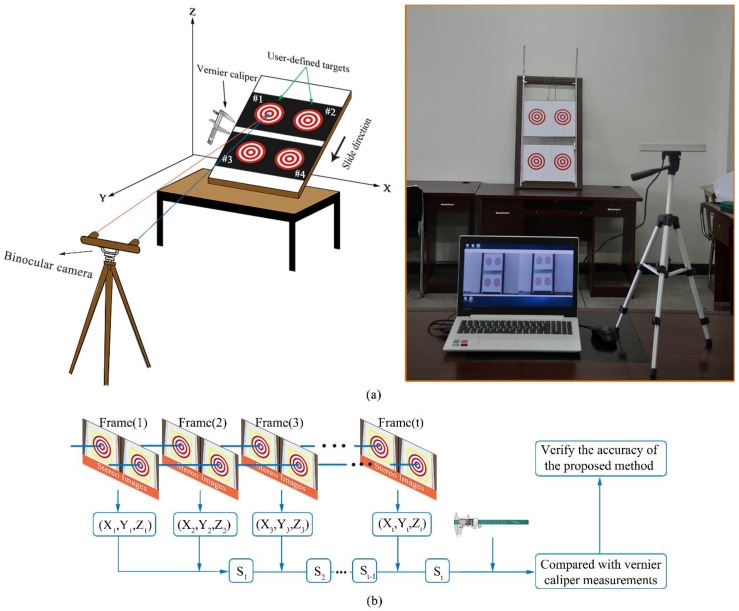
Moving platform experiment: (**a**) test site settings and (**b**) experimental procedure.

The above distance measurement test verifies the accuracy of the system proposed in this paper. However, the test capture process is static and cannot be used to verify the feasibility of the system. Based on this research, the laboratory model test is used to verify the tracking and positioning accuracy of the system. The overall layout of the test is shown in Figure 10. The test instruments included slidable panels, binocular cameras, Vernier callipers, and laptops. The slidable squad consists of two plates that can slide up and down, and can simulate the local deformation and overall deformation, respectively. To compare and analyse the accuracy, the sliding distance is obtained using the binocular stereo-vision system and Vernier calliper, respectively.

Test 1: Local deformation monitoring test. The lower plate is fixed in the sliding plate group, and the upper plate is moved slowly downwards. At the same time, a Vernier calliper and a binocular stereo-vision system are used to measure the distance between the two objects in the upper plate. A total of 34 frames are tested.

Test 2: Overall deformation monitoring test. The slope deformation is simulated by connecting the upper and lower plates, and moving them slowly at the same time. The displacement is quantified by monitoring the changes in the spatial position of the four landmarks. 

**Figure 11:**
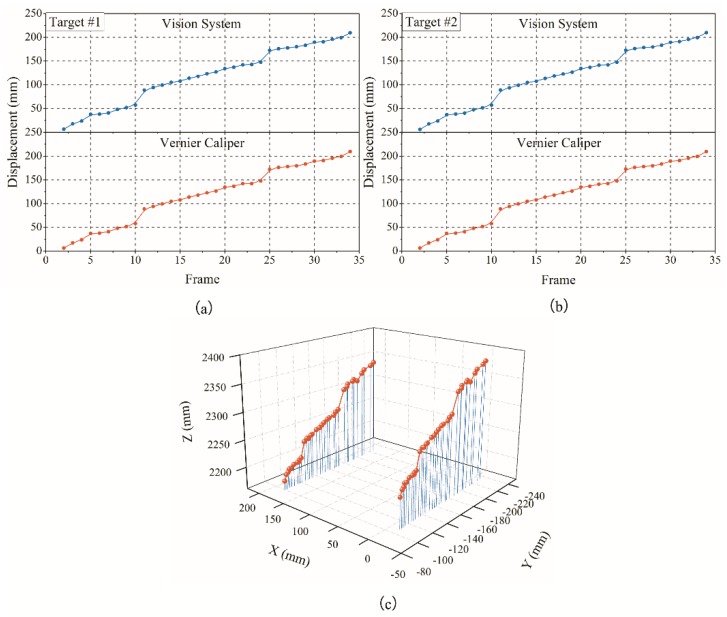
Local deformation marker point displacement monitoring results: (**a**) target 1, (**b**) target 2, and (**c**) spatial results.

**Figure 12:**
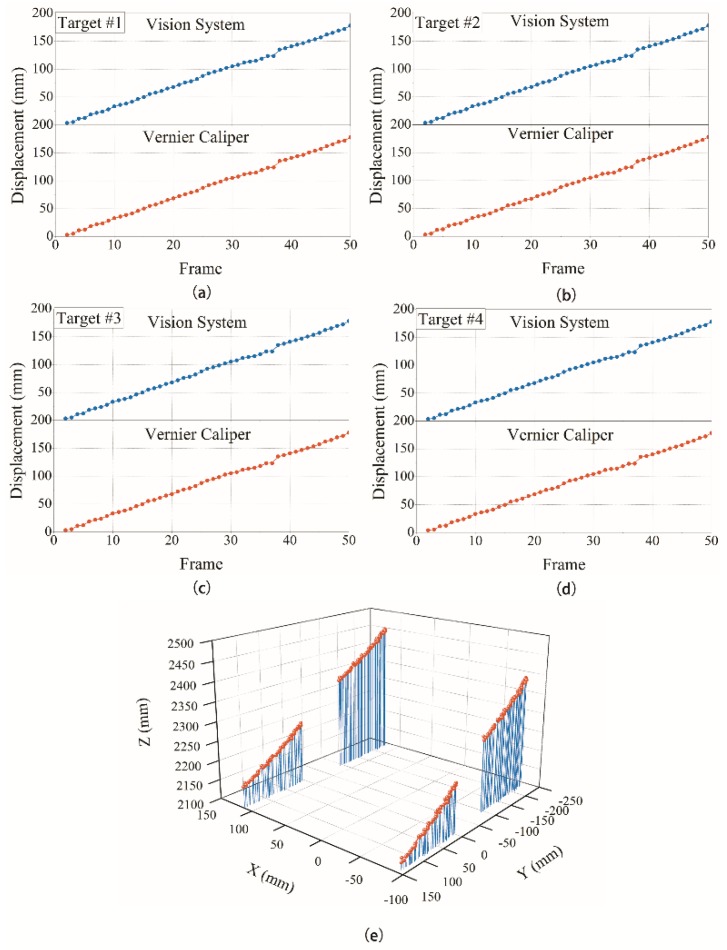
Overall deformation marker point displacement monitoring results: (**a**) target 1, (**b**) target 2, (**c**) target 3, (**d**) target 4, and (**e**) spatial results.

**Figure 13:**
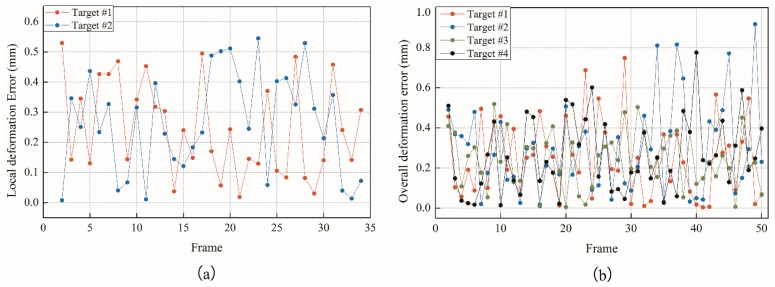
Results of measurement error: (**a**) Local deformation; (**b**) Overall deformation.

The test results obtained are shown in Figure 11, Figure 12 and Figure 13.

It can be seen from the above test data that the spatial displacement value tracked by the binocular stereo-vision measurement system is compared with the displacement value measured using the Vernier calliper. Through local deformation monitoring error can be obtained (see Figure 11 and Figure 13a), The average value of the error is 0.2568 mm, and the maximum error is 0.5427 mm. Only five of the 68 groups of measurement data have errors exceeding 0.5 mm, which proves that the binocular stereo-vision measurement system has strong tracking and positioning stability. The results of the overall deformation-monitoring test are shown in Figure 12 and Figure 13b. The average values of the monitoring errors for each marker are 0.2503, 0.2995, 0.2404, and 0.2619 mm, respectively, and the maximum error is 0.9219 mm. In the two hundred groups of stereo-vision system measurements, there are three groups with errors exceeding 0.8 mm, six groups with errors exceeding 0.7 mm, and eight groups with errors exceeding 0.6 mm. In addition, the mean and maximum values of the error are increased relative to the static measurement test. This is because there is a certain error in the target-tracking process, which causes the average error and fluctuation range to increase.

## 4. Conclusions

The exploration of structural health monitoring based on vision sensors is still in its infancy. In this study, a non-contact dynamic displacement measurement system with binocular stereo vision is designed. The slope is used as a carrier to explore the possibility of tracking and positioning technology to monitor the three-dimensional deformation of the structure. The specific conclusions are as follows:

(1) Target markers adapted to the monitoring system are specially designed as concentric circles. Considering the error of program operation, graphics positioning size and time cost, the research suggests setting the number of concentric layers to six, and the pixel size of the marker points to no smaller than 28 × 28 pixels. Under the design of the target, it can be seen from the noise robustness test that the positioning method has better positioning accuracy and stability under different levels of Gaussian noise and impulse noise than the center of gravity method.
(2) This study successfully introduces the target tracking technology into the deformation monitoring of the slope and improves the degree of intelligence. The tracking performance evaluation test shows that the use of UCC sub-pixel template matching technology to optimize the tracking accuracy of an STC target can effectively reduce the measurement error.
(3) Finally, slope movement is simulated by the indoor sliding plate, and the deformation is monitored employing the proposed method. The results show that the accuracy of the deformation measurement can achieve a millimeter level. It validates the potentials of the stereo vision displacement sensor for cost-effective slope health monitoring. However, the actual slope application needs to be further explored according to the actual situation.

The vision sensor system proposed in this paper can also be applied to deformation monitoring scenarios in other engineering fields, such as bridge deflection, tunnel convergence, and also structural deformation. However, detailed monitoring plans in these circumstances should take into full consideration specific site conditions and the primary monitoring objects closely related to the structural health.

## Figures and Tables

**Table 1:** Error analysis of different concentric circle tests.

Number of Circles	Measured Coordinates (pixel)		True Coordinates (pixel)		Error (pixel)		Time
	u	ν	u	ν	u	ν	(ms)
2	855.620117	855.671021	856	856	0.379883	0.328979	3707
4	855.615845	855.675781	856	856	0.384155	0.324219	3885
6	855.619934	855.677551	856	856	0.380066	0.322449	4134
8	855.619080	855.677856	856	856	0.380920	0.322144	4337
10	855.618164	855.677368	856	856	0.381836	0.322632	4477
12	855.611511	855.672791	856	856	0.388489	0.327209	4849
14	855.605957	855.669495	856	856	0.394043	0.330505	5184

**Table 2:** Concentric testing of different pixel dimensions.

Size (pixels)	Classify	Number of Circles Detected	Clustering Coordinates (pixels)		True Coordinates (pixels)		Error (pixels)	
			u	ν	u	ν	u	ν
41 × 41	circle	6	20.154753	20.214046	20.5	20.5	0.345247	0.285954
	ellipse	6	20.08853	20.192606	20.5	20.5	0.41147	0.307394
36 × 36	circle	6	17.625174	17.636324	18	18	0.374826	0.363676
	ellipse	6	17.594893	17.673111	18	18	0.405107	0.326889
30 × 30	circle	6	14.616336	14.700969	15	15	0.383664	0.299031
	ellipse	6	14.589076	14.743123	15	15	0.410924	0.256877
28 × 28	circle	6	13.625415	13.72232	14	14	0.374585	0.27768
	ellipse	6	13.612086	13.60793	14	14	0.387914	0.39207
25 × 25	circle	5	13.420197	11.999551	12.5	12.5	−0.9202	0.500449
	ellipse	6	12.436928	13.601745	12.5	12.5	0.063072	−1.10175

**Table 3:** Positioning error of marker points under the influence of noise.

Number	Noise Level	Gaussian Noise Error (pixels)				Impulse Noise Error (pixels)			
		Vision System		Gravity Method		Vision System		Gravity Method	
		u	ν	u	ν	u	ν	u	ν
1	0	0	0	0	0	0	0	0	0
2	0.005	0.002198	−0.00613	−0.00582	−0.00922	0.007202	0.000458	−0.06564	0.025549
3	0.01	0.000031	−0.00558	−0.01349	0.007616	−0.00336	−0.00061	0.094164	−0.01722
4	0.015	−0.000457	−0.00784	−0.0143	0.000161	−0.00513	−0.00552	0.087803	−0.0336
5	0.02	−0.006286	0.000122	−0.00445	0.024084	−0.00107	−0.00293	−0.00639	0.072873
6	0.025	0.002228	−0.00488	−0.01759	−0.00717	−0.00241	−0.00424	−0.05005	−0.11229
7	0.03	0.000641	−0.00748	0.001939	−0.00858	0.001587	−0.00052	0.008688	−0.01136
8	0.035	−0.002685	0.002228	−0.01136	−0.00445	0.000977	−0.00198	−0.00427	−0.13275
9	0.04	−0.004699	−0.00928	−0.01524	−0.00603	0.000977	−0.00598	0.016176	−0.08148
10	0.045	0.000489	−0.01071	−0.01459	−0.00226	−0.00095	−0.00345	0.09499	0.540542
11	0.05	0.003693	−0.00229	−0.0059	−0.00388	−0.00513	−0.00339	—	—
12	0.055	0.004456	−0.0101	−0.01893	−0.00204	0.005402	−0.00037	—	—
13	0.06	0.010865	−0.01099	−0.00166	0.006228	0.001984	−0.01364	—	—
14	0.065	0.002076	0.002808	−0.03957	−0.00365	−0.00018	0.004792	—	—
15	0.07	0.001526	−0.00134	0.000937	0.006315	0.002747	−0.01834	—	—
16	0.075	−0.009491	0.002289	−0.0064	−0.00334	0.004792	−0.00192	—	—
17	0.08	−0.002868	−0.00134	−0.00178	0.007308	−0.00082	0.002961	—	—

**Table 4:** Measurement results of Test 1.

Number	Minimum Diameter	Pixel Size	Target Space Coordinates			Measurement (mm)	Error (mm)
			x	y	z		
I-1	5	66 × 66	−47.2496	60.7972	1540.49	149.7765	0.2235
			101.466	64.0262	1522.99		
I-2	7.5	99 × 99	−45.7566	48.5814	1526.84	149.8464	0.1536
			102.9	55.1874	1544.49		
I-3	10	132 × 132	−72.0315	72.8357	1535.54	150.2367	0.2367
			76.8983	78.3795	1554.52		
I-4	12.5	165 × 165	−45.524	70.3383	1534.15	149.7905	0.2095
			101.925	71.9863	1507.82		
I-5	15	198 × 198	−33.9774	64.1482	1523.63	149.8362	0.1638
			115.1	67.5548	1538.3		
I-6	17.5	231 × 231	−84.4728	90.9581	1536.39	149.7684	0.2316
			63.3845	91.6196	1512.55		
I-7	20	264 × 264	−37.0946	87.539	1517.28	150.1354	0.1354
			108.986	84.4612	1482.76		
I-8	22.5	297 × 297	−101.026	85.8391	1518.59	149.8158	0.1842
			46.4737	86.6175	1492.36		

**Table 5:** Measurement results of Test 2.

Number	Real (mm)	Target Space Coordinates			Measurement (mm)	Error (mm)
		x	y	z		
II-1	100	−31.187	95.2045	1529.29	100.1681	0.1681
		66.6902	95.3143	1507.99		
II-2	125	15.1994	81.9168	1526.35	124.7468	0.2532
		139.581	85.3977	1535.23		
II-3	150	−33.9774	64.1482	1523.63	149.8362	0.1638
		115.1	67.5548	1538.3		
II-4	175	−84.989	109.588	1543.95	174.8049	0.1951
		87.5032	109.406	1515.61		
II-5	200	−36.0215	65.7006	1538.77	200.1552	0.1552
		163.315	67.5481	1556.76		
II-6	225	−86.3799	60.4379	1544.6	225.26	0.26
		137	61.2969	1573.63		
II-7	250	−65.7222	107.195	1540.87	250.1702	0.1702
		183.541	113.807	1561.1		
II-8	275	−52.424	74.3521	1516.97	275.2898	0.2898
		221.944	80.5141	1538.62		
II-9	300	−144.008	67.8541	1520.25	300.2821	0.2821
		154.401	74.3054	1553.11		
